# Pulmonary Arterial Hypertension: Recognition and Management in Primary and Acute Care Settings

**DOI:** 10.7759/cureus.100073

**Published:** 2025-12-25

**Authors:** Brian J Ahern, Sean T Fulford, Drew Long, Cheryl L Miller

**Affiliations:** 1 Emergency Department, William Beaumont Army Medical Center, Fort Bliss, USA; 2 Emergency Department, Madigan Army Medical Center, Joint Base Lewis-McChord, USA

**Keywords:** acute care, acute decompensation, emergency medicine, primary care, pulmonary arterial hypertension, pulmonary hypertension, right ventricular failure

## Abstract

Pulmonary arterial hypertension (PAH) is a rare but life-threatening condition marked by progressive elevation in pulmonary vascular resistance and right ventricular failure. Because symptoms, most commonly progressive exertional dyspnea, overlap with more common cardiopulmonary disorders, diagnosis is frequently delayed. Primary and acute care clinicians are often the first to evaluate these patients, placing them in a key position to recognize early clues and initiate appropriate evaluation. This review provides practical, evidence-based guidance for the frontline assessment of suspected PAH. Historical features such as exertional dyspnea out of proportion to known disease, unexplained fatigue, syncope, or chest discomfort should raise concern, particularly in patients with connective tissue disease or other risk factors. Initial evaluation involves common tests for evaluating cardiopulmonary complaints: electrocardiography, chest radiography, and measurement of B-type natriuretic peptide (BNP) or N-terminal pro-B-type natriuretic peptide (NT-proBNP). Transthoracic echocardiography is the most helpful initial imaging modality for estimating pulmonary artery pressures and assessing right ventricular structure and function. Definitive diagnosis, however, requires right heart catheterization, preferably at an accredited pulmonary hypertension (PH) center; empiric initiation of PAH-specific therapy is not recommended. By recognizing key clinical features, safeguarding ongoing therapy, and ensuring timely referral to PH centers, frontline clinicians may reduce diagnostic delays and improve outcomes for patients with PAH.

## Introduction and background

Pulmonary hypertension (PH) is present when mean pulmonary artery pressure exceeds 20 mmHg as measured by right heart catheterization (RHC) at rest, reflecting a hemodynamic state rather than a discrete disease [1]. It is classified into five groups based on underlying mechanisms and management strategies (Table 1)[1]. Among these, group 1 pulmonary arterial hypertension (PAH) is the most clinically distinct, characterized by progressive vasculopathy of the small pulmonary arteries and arterioles [2]. Remodeling and narrowing of these vessels drive rising pulmonary pressures, increased right ventricular strain, and ultimately right heart failure [3].

**Table 1:** Five clinical groups of pulmonary hypertension * Diagnosis confirmed by right heart catheterization, with each PH group showing characteristic patterns. † Schistosomiasis: leading global cause of PAH, uncommon in the US. ‡ Transplant (lung or heart–lung) is considered in advanced cases in groups 1 and 4. PH - pulmonary hypertension; CTD - connective tissue disease; CHD - congenital heart disease; ERA - endothelin receptor antagonist; PDE5i - phosphodiesterase-5 inhibitor; PGI₂ - prostacyclin analogue; sGC stim - soluble guanylate cyclase stimulator; CCB - calcium channel blocker; PAH - pulmonary arterial hypertension; COPD - chronic obstructive pulmonary disease; ILD - interstitial lung disease; CKD - chronic kidney disease Sources: [1,2]

Group*	Key association	Common conditions	Frequency	Treatment
Group 1: pulmonary arterial hypertension	Arteries → pulmonary arterial vasculopathy	Idiopathic, CTD, CHD, HIV, drug-related †	Rare	Targeted therapy (ERA, PDE5i, PGI₂, sGC stim); some respond to CCBs ‡
Group 2: PH due to left heart disease	Heart → left ventricular dysfunction	Heart failure, valvular disease, cardiomyopathy	Very common	Treat underlying heart disease; PAH meds not used
Group 3: PH due to lung disease/hypoxia	Lungs → chronic lung disease/hypoxia	COPD, ILD, sleep apnea, obesity hypoventilation	Common	Treat underlying lung disease; PAH meds are rarely used
Group 4: PH due to pulmonary artery obstruction	Clots → chronic obstruction	Chronic thromboembolic PH	Rare	Anticoagulation; endarterectomy; sGC stim if inoperable/residual ‡
Group 5: PH with unclear /multifactorial causes	Miscellaneous→ catch-all	Hematologic disease, sarcoidosis, CKD	Rare	Treat underlying disorder; PAH meds rarely indicated (expert only)

Although rare, PAH carries significant morbidity and mortality if not recognized early [3,4]. In the United States, about 95% of cases are either idiopathic or associated with underlying conditions, most often connective tissue diseases such as systemic sclerosis, lupus, or mixed connective tissue disease [5]. Symptoms such as exertional dyspnea, fatigue, chest pain, and edema are nonspecific and easily mistaken for more common disorders. As a result, PAH diagnosis is often delayed nearly two years from symptom onset, increasing the risk of irreversible right ventricular dysfunction and death [6,7].

Many patients with PAH are first seen in primary care, with some patients presenting to urgent care or emergency departments (EDs) with advanced disease [6,8,9]. The purpose of this review is to equip front-line clinicians with practical, evidence-based strategies for the recognition, evaluation, and initial management of suspected PAH in primary and acute care settings.

## Review

Clinical Clues to Suspecting PAH

Although non-specific, the earliest and most common symptom of PAH is dyspnea on exertion, often described as reduced exercise tolerance. Initially, patients may report shortness of breath only with strenuous activity, but as the disease and right ventricular dysfunction progress, dyspnea will occur with lighter activity or even at rest [2,3]. Fatigue, decreased stamina, and early exercise intolerance are common but nonspecific findings that frequently delay recognition. Presyncope and syncope, particularly with exertion, should raise concern for impaired right ventricular output and more advanced disease. Chest discomfort, palpitations, or unexplained episodes of lightheadedness are also frequently reported in later stages [6].

As PAH advances later in the disease process, clinical signs of right heart failure begin to emerge [10]. Peripheral edema, hepatomegaly, ascites, and weight gain from fluid retention are common late manifestations. Further examination and auscultatory findings may include elevated jugular venous pressure, a loud pulmonic component of the second heart sound, or a systolic murmur of tricuspid regurgitation [3]. These findings, though not always present, provide important bedside clues to the underlying hemodynamic burden.

Equally important is recognizing features that argue against PAH and suggest an alternative cardiopulmonary disorder [3]. Wheezing, significant cough, or sputum production are more consistent with obstructive lung disease such as asthma or chronic obstructive pulmonary disease (COPD). Orthopnea, paroxysmal nocturnal dyspnea, and prominent rales suggest left-sided heart failure rather than PAH. Fever, purulent sputum, or focal chest findings on auscultation point toward infectious etiologies. Similarly, acute pleuritic chest pain with hemoptysis and unilateral leg swelling should prompt consideration of pulmonary embolism as a primary diagnosis. Awareness of these overlapping but differentiating features helps prevent diagnostic anchoring and guides clinicians toward the most likely causes of a patient's presentation.

While symptoms such as dyspnea and fatigue are common across cardiopulmonary conditions, progressive exertional dyspnea, presyncope or syncope, and signs of right-sided heart strain should heighten suspicion for PAH, especially in patients with known risk factors such as connective tissue disease, familial PAH, and HIV [2]. Younger patients, as well as those with obstructive lung disease or obstructive sleep apnea, often experience longer delays in diagnosis compared with other groups [7]. Therefore, PAH should be considered in these populations when symptoms persist or progress despite optimal therapy. A thoughtful history and focused physical exam can narrow the differential and highlight patients who warrant further diagnostic evaluation.

Diagnostic Evaluation of PAH in Primary and Acute Care

The diagnostic evaluation of suspected PAH begins with the standard approach to cardiopulmonary complaints; However, this article focuses on PAH rather than a full workup of all causes of dyspnea. Patients in primary care who present with concerning vital signs, hypoxemia, or significant distress should be promptly referred to the ED for stabilization and further evaluation. At any point during the diagnostic process, if the clinician judges the probability of PAH to be high, patients should be referred to a PH center for further evaluation [1]. A list of US-accredited PH centers is available at https://phassociation.org/phcarecenters/accredited-centers/. When an in-person referral is not feasible, remote consultation with a PH center or referral to a local pulmonologist or cardiologist experienced in PH management is advised [11].

**Figure 1:**
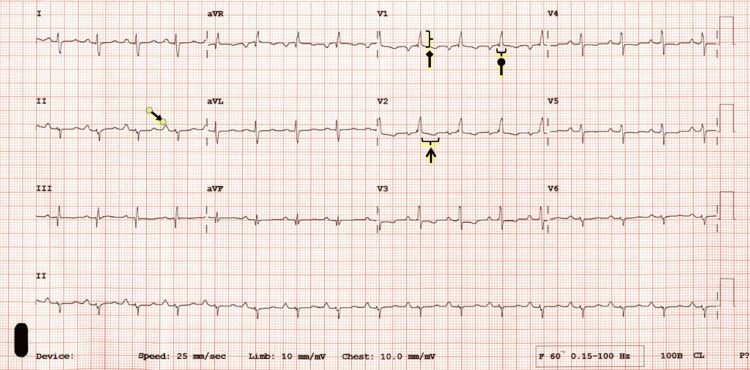
ECG demonstrating multiple abnormalities found in patients with pulmonary hypertension A: closed arrow shows P pulmonale (P>0.25 mV in lead II) B: circle arrow shows complete or incomplete right bundle branch block C: open arrow indicates RV strain pattern (ST depression; T wave inversion in the right precordial and inferior leads) D: diamond arrow indicates RV hypertrophy (R/S >1; R>0.5mV in V1; R in V1 + S in lead V5 > 1mV) Original image by authors.

Diagnostic testing for PAH should commence with a 12-lead electrocardiogram (ECG), chest radiograph, and laboratory studies on the initial encounter. A 12-lead ECG is a standard early test in patients with cardiopulmonary symptoms and can yield valuable clues in PAH. Always check for a prior ECG to assess for any acute changes. Typical ECG findings are shown in Figure 1 [3,6]. While ECG findings suggestive of PAH may be present in other cardiopulmonary conditions, they should raise suspicion in a patient with otherwise unexplained exertional dyspnea. In a single-center, retrospective study, eight of 61 PAH patients had a normal ECG, highlighting that a normal ECG does not rule out PAH [12].

A chest radiograph should be obtained in patients with suspected PAH, as well as in the broader evaluation of dyspnea and chest pain [1]. In PAH, common findings include enlargement of the central pulmonary arteries, pruning of the peripheral pulmonary vasculature, and right atrial or ventricular enlargement that changes the cardiac silhouette [6]. A chest radiograph is not diagnostic for PAH, but is helpful in identifying and suggesting other cardiopulmonary conditions, such as left heart failure, obstructive lung disease, pneumonia, or interstitial lung disease, that may better explain the patient's symptoms.

Laboratory assessment should include, but not be limited to, measurement of B-type natriuretic peptide (BNP) or N-terminal pro-B-type natriuretic peptide (NT-proBNP) [1,6]. These biomarkers provide biochemical evidence of right ventricular strain and can support the clinical suspicion of PAH. Elevated levels correlate with increased right heart pressures and worse functional status, but they lack specificity because they are also elevated in left heart failure and other causes of ventricular stress [13,14]. Conversely, normal BNP levels, particularly when paired with a normal ECG, are associated with a low likelihood of PAH, making them useful for identifying low-risk patients in whom alternative diagnoses may be more likely [1].

Pulmonary function testing (PFT) plays a supportive role in the evaluation of suspected PAH, helping distinguish PAH from PH due to chronic lung disease and identifying primary lung disorders [1]. Patients with PAH typically have normal spirometry and lung volumes but often demonstrate a mildly reduced diffusing capacity of the lung for carbon monoxide (DLCO). A severely low DLCO may be seen in PAH patients with systemic sclerosis [6]. In contrast, obstructive or restrictive patterns point to alternative causes of pulmonary hypertension, such as COPD or interstitial lung disease.

**Figure 2:**
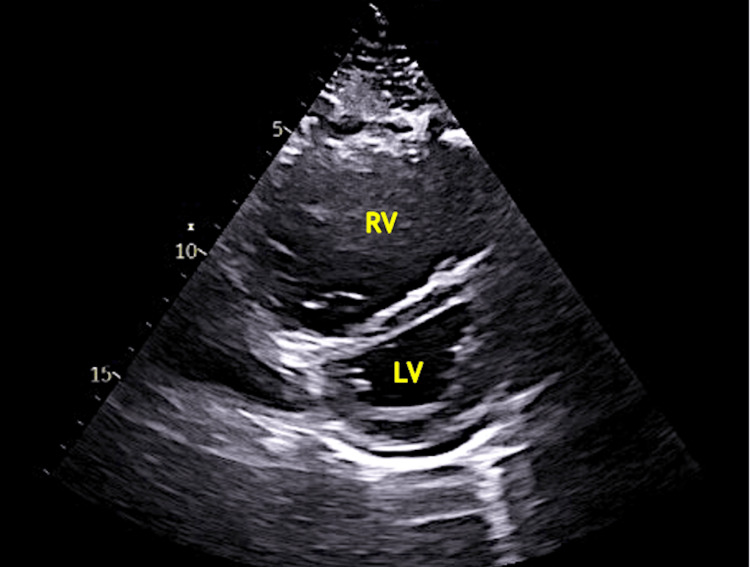
The parasternal short axis view demonstrates RV dilation with flattening of the interventricular septum due to increased RV pressures secondary to pulmonary hypertension RV - right ventricle; LV - left ventricle Original image by authors.

**Figure 3:**
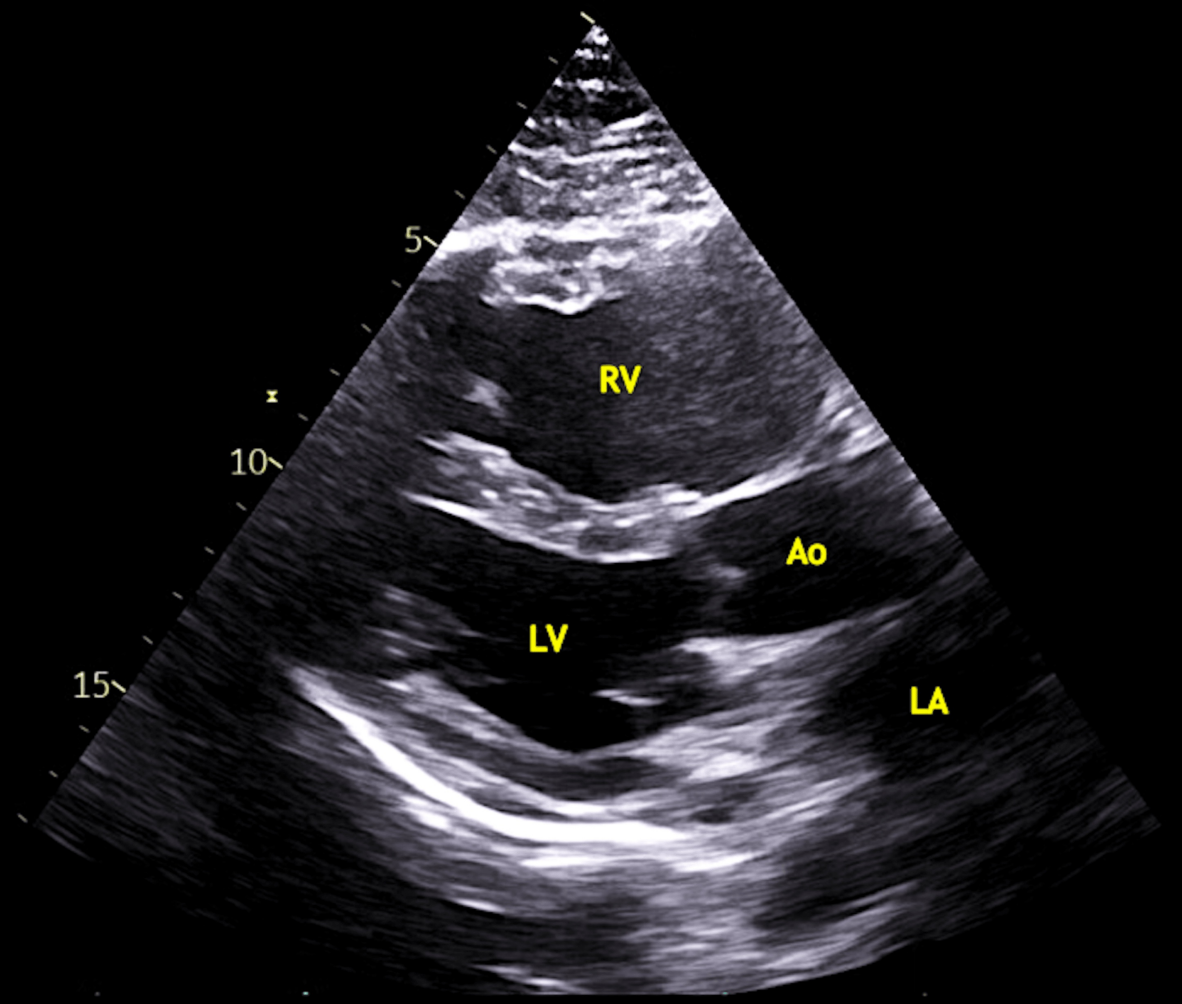
The parasternal long axis view again demonstrates right ventricle dilation with an RV/LV ratio >1 RV - right ventricle; LV - left ventricle; Ao - aorta; LA - left atrium Original image by authors.

Transthoracic echocardiography (TTE) is the most helpful initial noninvasive test for suspected PAH because it allows estimation of pulmonary artery pressures through measurement of the tricuspid regurgitant jet velocity [6]. Common findings in addition to tricuspid regurgitation include right atrial and ventricular enlargement, abnormal interventricular septal motion (Figures 2 and 3), and pericardial effusion, which often indicates advanced disease [3]. Point-of-care ultrasound can readily detect right heart strain or alternative diagnoses; however, a comprehensive echocardiogram is essential, as more detailed measurements and interpretation are required.

While TTE provides the clearest early assessment of PAH probability and severity, it cannot confirm the diagnosis; right heart catheterization (RHC) is required. Clinicians should be mindful that echocardiography may be limited by technical difficulties obtaining the requisite views and may also over- or underestimate pulmonary pressures [1]. In some cases, tricuspid regurgitation may be absent in patients with PH, which will further limit its diagnostic utility [3]. In most cases, RHC is warranted when the probability of PAH is high or intermediate in patients with known risk factors [1]. Conversely, when uncertainty remains after noninvasive testing, RHC may be reasonable even in patients with intermediate or low echocardiographic probability.

A correct diagnosis by RHC is essential because even when PAH is strongly suspected, empiric treatment is not recommended due to the potential for serious harm, such as hypotension. PAH-specific therapies are not indicated for other types of PH; therefore, inappropriate empiric treatment of PH with PAH-specific medications risks worsening left heart failure and other complications [2,3]. RHC directly measures mean pulmonary artery pressure and evaluates additional hemodynamic parameters critical for accurate diagnosis and classification. Each PH group demonstrates characteristic findings on RHC, which is ideally performed at the specialty center that will oversee ongoing management [6].

In summary, the evaluation of suspected PAH requires integrating findings from the history, exam, ECG, chest radiograph, biomarkers, echocardiography, and pulmonary function testing. No single test in the initial workup is diagnostic, but the combination of supportive results increases the likelihood of PAH. Conversely, a normal ECG together with normal BNP or NT-proBNP levels substantially lowers PAH probability. For those with a low likelihood of PAH and a more plausible alternative explanation for symptoms, further testing and treatment can be redirected accordingly. Ultimately, the PAH evaluation culminates with a definitive diagnosis made by RHC. 

Initial Management of Confirmed PAH

Management of PAH is highly specialized and best initiated through dedicated PH centers, with studies showing lower hospitalization rates and improved survival for patients managed by these centers [4]. Although primary or acute care clinicians do not typically start PAH treatment, it is important for them to recognize these medications and ensure that patients continue taking their PAH-specific therapies without interruption (Table 2) [9,10].

**Table 2:** Commonly used medications for pulmonary arterial hypertension CTEPH - chronic thromboembolic pulmonary hypertension; IV - intravenous; SC - subcutaneous; PDE5 - phosphodiesterase-5; PAH - pulmonary arterial hypertension Source: [10]

Drug class	Representative agents	Typical route(s)	Notes/indications
Prostacyclin analogues/receptor agonists	Epoprostenol, treprostinil	IV, SC, inhaled, oral	Potent vasodilators improve exercise capacity and outcomes. Treprostinil is available in multiple formulations.
Endothelin receptor antagonists (ERAs)	Ambrisentan, macitentan	Oral	Improve exercise capacity, delay clinical worsening; often used in upfront combination therapy.
Phosphodiesterase type-5 (PDE5) inhibitors	Sildenafil, tadalafil	Oral (IV for sildenafil)	Improve exercise capacity and delay clinical worsening; cornerstone of combination regimens.
Soluble guanylate cyclase (sGC) stimulator	Riociguat	Oral	Alternative to PDE5 inhibitors; indicated for PAH and inoperable/residual CTEPH.
Calcium channel blockers (CCBs) (in vasoreactive patients only)	Amlodipine, nifedipine	Oral	Reserved for a rare subset of PAH patients with positive vasoreactivity testing.

Management of PAH involves specialized medications that target different disease pathways. These include endothelin receptor antagonists, phosphodiesterase-5 inhibitors, soluble guanylate cyclase stimulators, and prostacyclin analogs [10]. A small subset of patients may benefit from calcium channel blocker therapy, with candidacy determined by vasoreactivity testing performed during RHC [3]. Combination therapy with multiple agents is generally more effective than single-drug treatment in reducing symptoms and slowing disease progression. High-risk patients with severe symptoms and elevated one-year mortality may be started on parenteral prostacyclin therapy delivered by a pump system by a PH center [15].

In addition to disease-specific therapies, several supportive measures are important in managing patients with PAH. Diuretics can help relieve volume overload and ease symptoms of right heart failure. Data on supplemental oxygen in PAH is limited; however, it is recommended for patients with persistent resting hypoxemia (PaO_2_ less than 60 mm Hg) [3]. For patients traveling to high altitudes, oxygen should be used to maintain saturations above 91%. Elective surgeries, if necessary, are safest when performed at specialized PH centers [11]. Counseling women of childbearing age about pregnancy is also essential, as pregnancy carries a high risk of both maternal and fetal complications in PAH [3,10]. When contraception is indicated, estrogen-containing methods should be avoided because of their association with venous thromboembolism [3].

Acute Decompensated PAH

Acute decompensation in PAH is typically driven by acute right heart failure, most often precipitated by infection, arrhythmia, uncontrolled volume status, medication interruption, or pulmonary embolism [16,17]. For patients with known PAH presenting with decompensation, early consultation with a PH center is critical to assist with management and facilitate transfer, if needed [18]. Patients often present with increased work of breathing, tachycardia, hypotension, peripheral edema, and jugular venous distension, findings that reflect the failing right ventricle's inability to maintain forward flow [19]. Even with resuscitation and critical care, in-hospital mortality exceeds 35% [18].

Missed or interrupted PAH therapy can precipitate acute decompensation; therefore, ensuring continuity of these medications is critical in the ED and hospital settings [9,20]. Abrupt discontinuation of subcutaneous or intravenous prostacyclin infusions can cause rapid rebound PH with resultant right heart failure and hypotension [21,22]. Clinicians should verify adherence, resume home regimens promptly, and provide stop-gap prescriptions or inpatient dosing when necessary. Special caution must be exercised with drug interactions: nitrates such as nitroglycerin and isosorbide mononitrate are absolutely contraindicated in patients on phosphodiesterase-5 inhibitors or guanylate cyclase stimulators due to the risk of catastrophic hypotension [23]. Counseling patients to alert EMS and other clinicians of this contraindication is a practical step to reduce iatrogenic harm.

In the ED, decompensated PAH management is time-sensitive and begins with rapid identification of PAH and reversal of precipitating factors. Acidosis, hypoxia, and hypercapnia all increase pulmonary vascular resistance and worsen PAH, so resuscitation and optimization of hemodynamics are crucial [24]. Supplemental oxygen should be provided for hypoxemia. While intubation with mechanical ventilation may be necessary in cases of respiratory failure, it should be approached very cautiously, as induction medications for intubation and positive-pressure ventilation can rapidly worsen right ventricular function and lead to cardiovascular collapse [25]. High-flow nasal cannula is a useful option to improve oxygenation without the risks associated with intubation and has not been shown to adversely affect right heart function in stable or critically ill patients with PH [26-28]. 

If intubation is required in a patient with PAH, hemodynamic optimization before induction and initiation of positive-pressure ventilation is essential. Preload should be carefully balanced and titrated: diuretics may be used to relieve volume overload, while small crystalloid boluses of approximately 250 mL can correct dehydration. Large fluid boluses, however, should be avoided as they can worsen right heart failure [29]. In patients with right ventricular failure, expert consensus recommends avoiding systemic hypotension, generally maintaining a mean arterial pressure (MAP) above 60 mm Hg, or a MAP capable of maintaining end-organ perfusion [30-33]. When MAP is below this target, vasopressors are indicated; norepinephrine is preferred as the first-line agent to maintain systemic perfusion, with vasopressin as a suitable second-line option because it does not increase pulmonary vascular resistance [16,18,20]. Compared with norepinephrine and vasopressin, phenylephrine is generally considered suboptimal since it raises afterload without enhancing contractility [9,30]. If additional inotropic support is needed after achieving target MAP, dobutamine is preferred, with milrinone as an alternative [33]. In refractory cases of decompensated PAH, extracorporeal membrane oxygenation (ECMO) can be considered in consultation with PH experts as a bridge to lung transplantation or recovery, though outcomes remain guarded, with nearly half of such patients not surviving hospitalization [18].

## Conclusions

PAH is a rare but life-threatening condition that often mimics more common cardiopulmonary diseases, leading to diagnostic delays. For primary and acute care clinicians, the key responsibilities are recognizing when to suspect PAH, conducting a focused initial evaluation without overlooking more common conditions, and referring for definitive diagnosis with RHC at a PH center. Practical bedside findings, ECG, chest radiograph, biomarkers, echocardiography, and pulmonary function testing help estimate disease probability. Continuity of PAH-specific therapy is critical, as missed doses or harmful drug interactions can cause rapid decompensation. Acute decompensated PAH warrants hospital admission and is associated with high mortality. By maintaining vigilance, ensuring timely referral, and safeguarding ongoing therapy, front-line clinicians play a decisive role in reducing delays and improving outcomes for patients with PAH.
